# Cell Type-Specific Responses to Wingless, Hedgehog and Decapentaplegic Are Essential for Patterning Early Eye-Antenna Disc in *Drosophila*

**DOI:** 10.1371/journal.pone.0121999

**Published:** 2015-04-07

**Authors:** Jong-Hoon Won, Orkhon Tsogtbartarr, Wonseok Son, Amit Singh, Kwang-Wook Choi, Kyung-Ok Cho

**Affiliations:** 1 Department of Biological Sciences, Korea Advanced Institute of Science and Technology, 291 Daehak-ro, Yuseong-gu, Daejeon, Korea; 2 Department of Biology, Center for Tissue Regeneration and Engineering at Dayton (TREND), University of Dayton, 300 College Park, Dayton, Ohio 45469-2320, United States of America; University of Massachusetts Medical School, UNITED STATES

## Abstract

The *Drosophila* eye-antenna imaginal disc (ead) is a flattened sac of two-layered epithelia, from which most head structures are derived. Secreted morphogens like Wingless (Wg), Hedgehog (Hh), and Decapentaplegic (Dpp) are important for early patterning of ead, but the underlying mechanisms are still largely unknown. To understand how these morphogens function in the ead of early larval stages, we used wg-LacZ and dpp-Gal4 markers for the examination of wild-type and mutant eads. We found that the ead immediately after hatching was crescent-shaped with the Bolwig’s nerve at the ventral edge, suggesting that it consists of dorsal domain. In a subsequent step, transcriptional induction of *dpp* in the cells along the Bolwig’s nerve was followed by rapid growth of the ventral domain. Both Wg and Hh were required for the formation of the ventral domain. Wg was crucial for the growth of the entire ead, but Hh was essential for cell division only in the dorsal domain. In the ventral domain, Hh regulated *dpp* transcription. Based on these data, we propose that signaling among distinct groups of cells expressing Wg, Dpp, or Hh in the ead of the first-instar larvae are critical for coordinated growth and patterning of ead.

## Introduction

All adult structures are developed from primordia that are initiated from a small number of founder cells whose proliferation and differentiation are controlled by multiple signaling molecules and domain-specific selector proteins [1,2]. Imaginal discs in *Drosophila melanogaster* have been an excellent system for studying this developmental process. Among imaginal discs, the eye-antenna disc (ead) contains primordia for eye, antenna, ocelli, palpus and head cuticles [3–5]. Morphogens such as Hedgehog (Hh), Wingless (Wg), and Decapentaplegic (Dpp) are important for growth and regional specificity of ead including the initial patterning of the first-instar (L1) ead [6–13]. However, little is understood how ~20 cells of L1 ead respond to these morphogens for their growth and patterning [11].

Imaginal discs have flattened sac-like structures of a continuous epithelial sheet: the upper and the bottom epithelial layers of an imaginal disc are called peripodial epithelium (PE) and disc proper (DP), respectively [14–18]. PE cells are easily distinguished from DP cells by their large size and squamous shape, especially in third-instar (L3) eads [11]. In terms of growth, PE and DP cells need to proliferate coordinately in order to maintain the flattened disc morphology. Electron microscopic analyses uncovered the presence of growth coordination in DP and PE [19], and several groups subsequently reported that DP and PE layers are involved in inter-epithelial signaling [11,20–22]. In terms of patterning, it has been shown that Wg and Dpp are expressed in the dorsal and ventral domains of the PE, respectively, while Hh is expressed in both layers of ead. Dorsal and ventral domains of PE have been defined based on the specific expression of Wg and Dpp in the PE of L1 ead. In DP, Delta and Serrate are preferentially expressed in the dorsal and ventral domains, respectively [11]. However, it has not been studied whether the DV domain is already present in the L1 ead from the beginning.

Expression patterns of the L1 ead suggest that the domains in PE and DP may have different embryonic origin. Indeed, several reports support this view. First, ead is generated by the fusion of six embryonic head segments [17,23,24], unlike wing and leg discs that are composed of cells from a single thorax segment [16]. Second, the outer and inner layers of the dorsal pouch are originated from different embryonic segments, and form the PE and the DP, respectively [3,17,25]. Third, *wg* and *ocelliless (oc)/orthodenticle* (*otd*) are expressed in the same region of dorsal domain in the L2 ead, suggesting that *wg* and *oc* are expressed in the cells with the same embryonic origin [8]. Thus, it is reasonable to speculate that cells from a segment would show specificity in their expression of morphogens as well as in their response to the morphogens, although presence of multiple embryonic segments in the ead has made the boundary obscure and difficult to visualize.

We have previously reported the expression pattern of cells expressing Wg, Hh or Dpp in the L1 and L2 eads using LacZ markers [11]. One of the limitations of that work was that these LacZ markers could not be visualized in the same disc. Here, we examined the Wg- and Dpp-expressing cells in the same specimen using two markers, wg-LacZ reporter in *wg* ^*en11*^ allele [26] and GFP marker expressed by *dpp-Gal4*.*PS* driver [27] to address the following questions. 1) What is the domain structure of the L1 ead right after hatching? 2) Are there different types of cells in the L1 ead? 3) What are the distinct functions of Hh, Wg and Dpp in the patterning and growth of early eads? 4) Can cells in the L1 ead be correlated with those in the L3 ead? We examined for the first time the expression pattern of L1 and L2 eads in detail, and have shown that different cell types in the L1 ead exhibit distinct responses to morphogens in their growth and patterning.

## Materials and Methods

### Fly Stocks and crosses

*wg* ^*en-11*^ */CyO* line containing a lacZ reporter inserted in the *wg* gene was used to mark the cells expressing Wg (wg-LacZ^+^ cells) [26], and *UAS-GFP* expressed by *dpp-Gal4* driver was used to examine the cells expressing Dpp by *dpp-Gal4*.*PS* driver (dpp-Gal4^+^ cells) [27]. The level of Wg protein was highest in the wg-LacZ^+^ cells, and was gradually decreased in neighboring cells (S1 Fig.), indicating that wg-LacZ is a reliable marker for Wg-producing cells.

In case of *wg* mutation, we used heteroallelic combination of *wg* ^*en-11*^, an amorphic allele, and *wg* ^*I-12*^, a temperature-sensitive allele [28]. The *wg* ^*I-12*^ */wg* ^*en-11*^ mutants never reached pupal stage. To obtain *wg* ^*I-12*^ */ wg* ^*en-11*^; *UAS-GFP/ dpp-Gal4* larvae, *wg* ^*I-12*^ */CyO-GFP; UAS-GFP* flies were crossed with *wg* ^*en-11*^ */CyO-GFP; dpp-Gal4 /TM6 Tb* flies. After egg collection for one day and culture for one more day at room temperature, the progeny was shifted to 30^°^C for 1 day, shifted back to room temperature, and then *wg* ^*I-12*^ */wg* ^*en-11*^; *dpp-Gal4 /UAS-GFP* larvae were dissected. Tissues were stained to visualize LacZ, GFP and Discs-Large (Dlg) [29].

In case of *hh* mutation, embryos were collected from *wg* ^*en-11*^ */CyO; hh* ^*ts2*^ */TM6 Tb* flies for one day and cultured one or two days at room temperature, and then shifted to the restrictive temperature (30^°^C) until dissection. *Tb* ^*+*^ larval progeny that are homozygous for *hh* ^*ts2*^ were dissected. To examine the effects of overexpression of CycE or P35 on the pattern of Wg- and Dpp-expressing cells in *hh* ^*ts2*^ background, the following two-step crosses were carried out. First, *wg* ^*en-11*^ */CyO; D/TM6 Tb* were crossed with *dpp-Gal4 hh* ^*ts2*^ */TM6 Tb* and the progeny *wg* ^*en-11*^ */+; dpp-Gal4 hh* ^*ts2*^ */TM6 Tb* females were obtained. These *wg* ^*en-11*^ */+; dpp-Gal4 hh* ^*ts2*^/TM6 female progeny were crossed with three different kinds of males: (i) *hh* ^*ts2*^ *UAS-GFP/TM6 Tb*, (ii) *UAS-CycE; hh* ^*ts2*^ *UAS-GFP/TM6 Tb*, (iii) *UAS-P35; hh* ^*ts2*^ *UAS-GFP/TM6 Tb*.

In case of *dpp* mutation, *wg* ^*en-11*^ *dpp* ^*d14*^ */CyO-GFP; dpp-Gal4/TM6 Tb* females were crossed with *dpp* ^*d-blk*^ */CyO-GFP; UAS-GFP*. Among the progeny, *wg* ^*en-11*^ *dpp* ^*d14*^ */ dpp* ^*d-blk*^; *dpp-Gal4/ UAS-GFP* were used for analysis.

### Generation of GFP clones

To generate GFP clones, *UAS-GFP* males and *y w P[actin>CD2>Gal4; w* ^*+*^ *]; hsFlp*, *MKRS/TM6 Tb* females were crossed. Their progeny during the late embryonic and the early L1 stage were heat-shocked at 37^°^C for 1 hr, and then cultured at room temperature until dissection [30]. To generate GFP clones in *wg-LacZ* background, *wg* ^*en-11*^ */CyO; UAS-GFP* males were used for crossing instead of *UAS-GFP* males.

### Immunocytochemistry

Dissected imaginal discs were stained with fluorescent markers [31]. Primary antibodies used in this study were mouse anti-β-galactosidase (Promega; 1:250), rabbit anti-β-galactosidase (Cappel; 1:200), chicken anti-GFP (Upstate; 1:100), rabbit anti-GFP (Biogenesis; 1:100), rabbit anti-Dlg (1:1,000) [32], 22C10 (DSHB; 1: 150), mouse anti-Wg (DSHB; 1:100). Secondary antibodies conjugated with Cy3, Cy5, fluorescein isothiocyanate were purchased from Jackson Laboratories. Fluorescent images were captured using a Zeiss laser-scanning confocal microscope and processed with Adobe Photoshop. Only a few representative images were picked for generating figures, and a complete set of confocal sections were shown in supplementary figures in certain cases.

## Results

### The early L1 ead has only dorsal domain

**Fig 1:**
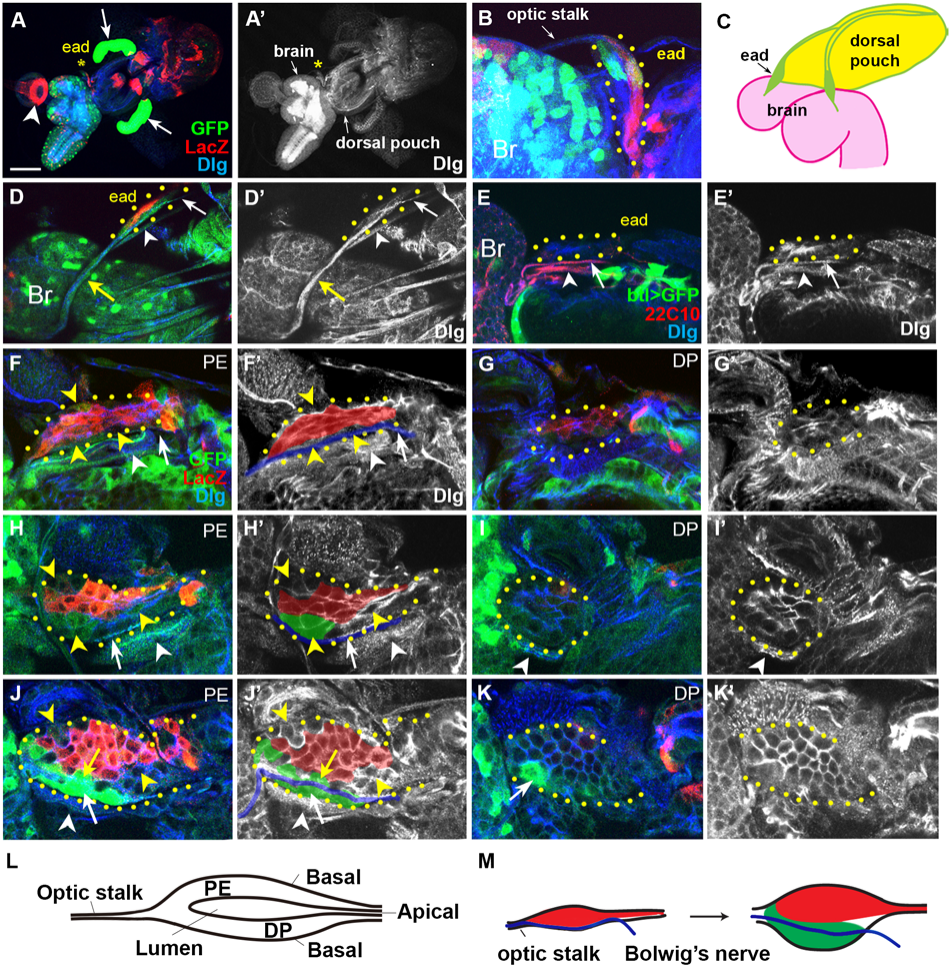
Growth of the ead from hatching to mid-L1 stage. Cell boundary visualized with anti-Dlg antibody is marked with either blue (A-K), or white (A’-K’). wg-LacZ^+^ and dpp-Gal4^+^ cells are marked with red and green, respectively except (E). Confocal images of PE, DP, and both combined (COM) are indicated in upper right. Boundary of the ead is marked with yellow dots. (A, A’) Arrow and arrowhead in (A) indicate salivary gland and proventriculus, respectively. The eads are marked with asterisks. (B) Same sample shown in (A) is magnified to show the ead enclosed in a yellow-dotted circle. (C) A diagram of ead, brain, and dorsal pouch of the L1 ead. (D) Two nerve-like structures on the ventral side of the newly hatched early L1 ead. Arrow and arrowhead indicate Bolwig’s nerve and the unknown nerve, respectively. Yellow arrow points the optic stalk and the Bolwig’s nerve within. The sample is unusually stretched during preparation, making the two nerves easily distinguishable. (E) The Bolwig’s nerve (arrow) and the unknown nerve (arrowhead) are marked with a neuron-specific marker 22C10, not marked with GFP expression driven by a trachea-specific breathless-Gal4. (F, G) PE (F) and DP (G) layers of the ead from a newly hatched larva. Bolwig’s nerve is marked with white arrows (F, F’) and blue line (F’). (H, I) PE (H) and DP (I) layers of a little older ead. (J, K) A mid-L1 ead with cells expressing high level of dpp-Gal4 on the dorsal (yellow arrow in J) or ventral (white arrow in J) side of the Bolwig’s nerve (blue line in J’). (L) A cross-section of L1 ead is diagrammed. Apical surfaces of the PM and DP layers face each other in the disc lumen. (M) A diagram showing the formation of new ventral domain and the position of Bolwig’s nerve during this stage on the PE layer. Scale bar: A, 100 μm; B, D-E, 20μm; F-K, 10 μm.

The L1 ead is a tiny sac with two-layered epithelium attached to the posterior end of dorsal pouch, and is formed by evagination from dorsal pouch during late embryogenesis [23]. Despite its tiny size, the ead of newly hatched larvae was readily recognized by its distinct location between the dorsal pouch and brain as well as the presence of optic stalk that links the ead and the brain (Fig. 1A-C). We found two nerve-like structures on the ventral side of the early L1 ead (Fig. 1D). One of them was the Bolwig’s nerve, which was connected to PE at the anterior ventral region and entered the brain through the optic stalk (white arrows in Fig. 1D; S2 Fig.), and the other was an unknown nerve whose axon entered the central brain (arrowhead in Fig. 1D and S2 Fig.). These nerve-like structures were visualized with 22C10 antibody that specifically recognizes Futsch/22C10 (Fig. 1E), a neuronal MAP12B-like protein [33], and did not express GFP driven by breathless-Gal4 [34,35]. This demonstrated that the nerve-like structures are indeed nerves but not trachea (Fig. 1E and S3 Fig.). Two nerves were easily distinguishable because the Bolwig’s nerve was connected to the PE layer (S2F Fig.) while a branch of the other nerve was connected to the DP layer (S2G Fig.). The location of the Bolwig’s nerve along the ventral edge of the early L1 ead was a contrast to that along the DV midline of the PE in the late L3 ead [36–38].

The ead of the newly hatched larvae was crescent-shaped and had ~20 cells, 10 in PE and DP, respectively (Fig. 1F, G and S4 Fig.). The size of PE and DP cells was not much different in the L1 ead (Fig. 1F’, G’), unlike the L3 ead [11]. Most PE cells expressed wg-LacZ marker (referred as ‘wg-LacZ^+^ cells’) (Fig. 1F), but a small number of cells on the dorsal and ventral edges did not express wg-LacZ (yellow arrowheads in Fig. 1F, F’). Bolwig’s nerve itself exhibited a low level of dpp-Gal4 expression (Fig. 1D and S2F Fig.). In a subsequent stage, PE cells expressing dpp-Gal4 at a low level (referred as ‘dpp-Gal4^+^ cells’) were observed between the Bolwig’s nerve and the wg-LacZ^+^ cells (Fig. 1H and S5 Fig.), which may be daughter cells of the cells on the ventral edge described in Fig. 1F (yellow arrowhead). Cells that did not express wg-LacZ were also increased in number on the dorsal edge and anterior region (yellow arrowheads in Fig. 1H).

### Ventral domain is formed in the mid-L1 ead

**Fig 2:**
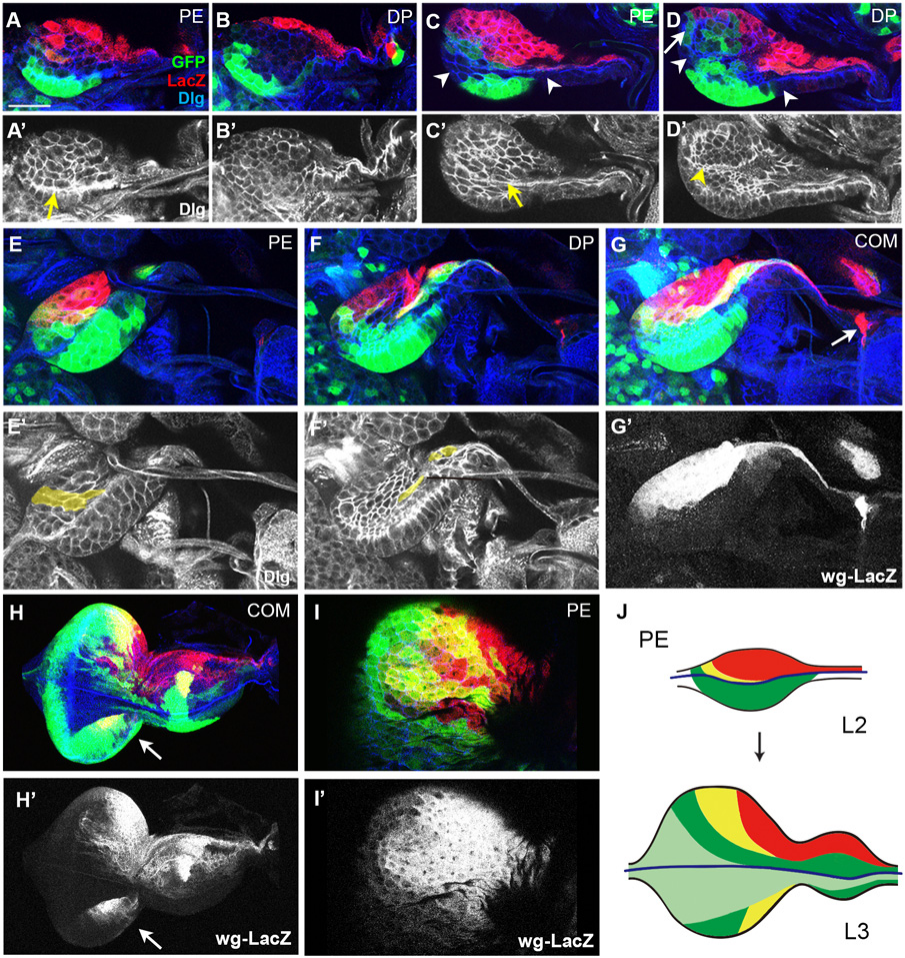
Growth of the ead from the late L1 to the mid-L3 stage. Cell boundaries visualized with anti-Dlg antibody are shown in A’-F’. (A, B) Late L1 ead. Bolwig’s nerve is marked with a yellow arrow in A’. (C, D) Bolwig’s nerve in PE (C, C’) and a groove in DP (D, D’) layers are marked with yellow arrow and arrowhead in an early L2 ead, respectively. The white arrow indicates dorsal dpp-Gal4^+^ cells in DP. (E-G) Midline cells (yellow) in PE (E) and DP (F) during mid-L2 stage are painted yellow in E’ and F’. Combined image shows dorsal pouch as the origin of wg-LacZ^+^ cells in G (arrow). wg-LacZ pattern is shown in G’. (H) A mid-L3 ead. Another new wg-LacZ^+^ cells are appeared on ventral region in H (arrow). (I) Dorsal PE region in (H) is magnified, and black and white images of wg-LacZ (H’, I’) are shown. (J) A diagram showing the changes in PE layer of ead during L2 and L3 stages. Scale bar: A-G, 20 μm; H, 90 μm; I, 50μm.

Some cells that express dpp-Gal4 at a high level appeared ventral to the Bolwig’s nerve for the first time in mid-L1 eads (Fig. 1J and S6 Fig.). In addition, some PE cells juxtaposed the dorsal side of the Bolwig’s nerve (yellow arrow in Fig. 1J) started to express dpp-Gal4 marker at a high level. These dpp-Gal4^+^ cells in PE of the late L1 ead, especially in the ventral side, were increased in number (Fig. 2A and S7 Fig.). As a result, the ead with balanced dorsal and ventral domains was eventually formed in the mid-L2 stage (Fig. 2C). Small number of cells that did not express dpp-Gal4 was also present in the ventral PE and DP (arrowheads in Fig. 2C, D), suggesting that different cell types participated in the formation of ventral domain.

In contrast to the dynamic expression of PE cells, DP cells expressed Wg and Dpp at a low level until mid-L1 stage (Fig. 1G, I). In mid-L1 ead, a cell in ventral DP expressed dpp-Gal4 at a high level (arrow in Fig. 1K). This cell may be one of founder cells that become a major cell type in ventral DP in the later stages (Fig. 2B, D). A groove appeared in the DV midline of the DP in mid-L2 ead (arrowhead in Fig. 2D’), which may be the origin of the equatorial groove occasionally seen in L3 eye disc [39,40]. After establishment of the ventral domain, a new group of cells became visible in the midline region of the mid-L2 ead (Fig. 2E-G). These cells, referred as ‘midline cells’, may be a ventral part of dorsal wg-LacZ^+^ cells, which increase the expression of dpp-Gal4 at this stage (shaded yellow in Fig. 2E’, F’).

Asynchronous formation of dorsal and ventral domains suggests that the dorsal cells are clonally distinct from the ventral cells. To address this point, we generated GFP clones by FLP-FRT method during late embryonic and early L1 stages and then examined them in the late L3 stage [41]. Although GFP clones generated in the DP were excluded from this analysis because of extreme complexity (data not shown), GFP clones in the PE were consistently present in anterior-posterior direction but not in dorsal-ventral direction (S8 Fig.). This supports the idea that the dorsal domain is clonally distinct from the ventral domain in PE. Furthermore, multiple GFP clones occupied different regions of the ventral PE, suggesting that the ventral PE is also the composite of cells formed from multiple founder cells (S8C-F Fig.).

We also examined GFP clones near the Bolwig’s nerve in order to check whether the Bolwig’s nerve is a clonal boundary in PE (S8A-D Fig.). These GFP clones trespassed on the Bolwig’s nerve, but were always present in anterior-posterior direction. This is still consistent with our finding that dorsal domain is formed before the ventral domain. Actually, this trespassing reminded of the dpp-Gal4^+^ cells that were present on both dorsal and ventral sides of the Bolwig’s nerve in the mid-L1 ead (compare yellow and white arrows in S8B, D Fig. to yellow and white arrows in Fig. 1J). Thus, we propose that the Bolwig’s nerve is not a strict clonal boundary but still a reliable DV boundary in PE.

### Position of various cell types in early eads is maintained during larval stages

At least four cell types that express Wg and Dpp differentially were present in the PE of L2 ead: dorsal wg-LacZ^+^ cells, dorsal dpp-Gal4^+^ cells, midline cells, and ventral dpp-Gal4^+^ cells, and other cells that express neither wg-LacZ nor dpp-Gal4 were also present. These cells maintained their relative positions in the L3 ead (Fig. 2H, I and S9 Fig.). Ventral wg-LacZ^+^ cells additionally appeared later in L3 ead (arrows in Fig. 2H, H’). Since the wg-LacZ^+^ cells in dorsal PE are one of the oldest cell types in ead, we examined whether these cells are clonally distinct by comparing the randomly generated GFP clones and wg-LacZ pattern. One ead had two independent GFP clones, one near the midline and the other on the dorsal edge, with peripodial wg-LacZ^+^ cells between them (S10A, B Fig.). In another ead, a GFP clone was directly juxtaposed to wg-LacZ^+^ cells on the basal region of ead that was continuous from the dorsal wg-LacZ^+^ cells (S10C, D Fig.). Thus, these dorsal wg-LacZ^+^ cells in PE are clonally distinct from the neighboring cells, and the ead may consist of cells with multiple origins.

### Wg is essential for the growth of L1 ead

**Fig 3:**
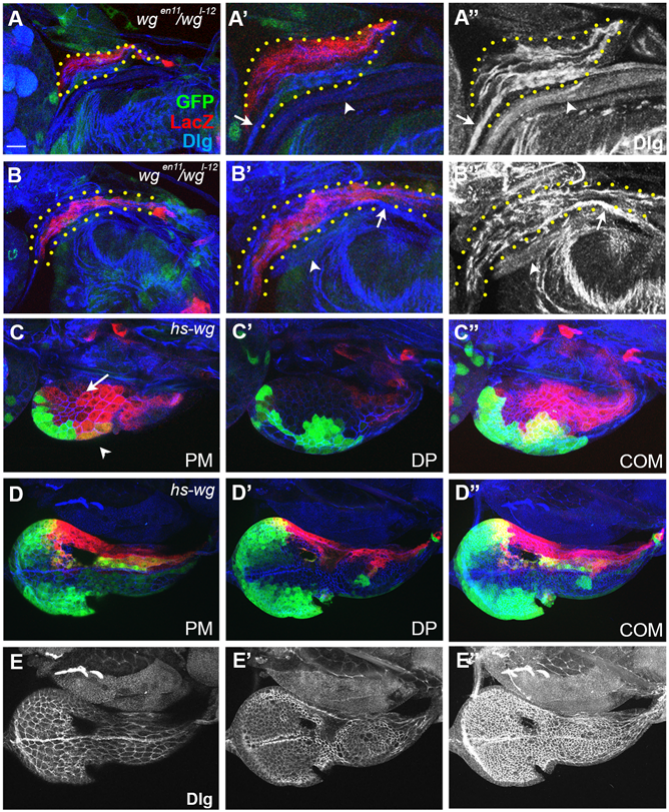
Wg is important for the growth of ead and the establishment of the ventral domain. A and B were magnified 2 times in A’ and B’. Cell boundaries visualized with anti-Dlg antibody are shown in A”, B” and E. (A) *wg* ^*I-12*^ */ wg* ^*en-11*^ ead with no ventral domain. Bolwig’s nerve and the unknown nerve are marked with arrow and arrowhead, respectively. (B) An older ead from a *wg* ^*I-12*^ */ wg* ^*en-11*^ larva after prolonged culture. (C) *hs-wg* ead. (C”) is a combined image of (C’, PE) and (C, DP). (D, E) Some midline (arrow) and ventral wg-LacZ^+^ (arrowhead) cells are missing in *hs-wg* ead. (E-E”) The images of Dlg in (D-D”) show the cell boundary and Bolwig’s nerve. Scale bar, 10 μm.

We have previously reported that Hh, Dpp, and Wg are essential for the growth and patterning of early eads [11]. To study how the cell types are influenced in the mutant eads, we analyzed the pattern of wg-LacZ^+^ and dpp-Gal4^+^ cells in the ead of hypomorphic or strong temperature-sensitive *wg*, *hh*, and *dpp* mutants since null mutants of these genes are embryonic lethal. The heteroallelic *wg* ^*I-12*^ */ wg* ^*en-11*^; *UAS-GFP/ dpp-Gal4* larvae were very small and thin even after prolonged culture at room temperature, and never reached pupal stage. The *wg* mutant eads had a thin strip of wg-LacZ^+^ cells but no ventral domain (Fig. 3A). The *wg* ead from older larvae also exhibited the similar phenotype (Fig. 3B). In all *wg* mutant eads examined, midline cells and dorsal dpp-Gal4^+^ cells were absent. These suggest that Wg is crucial for the formation of the ventral domain as well as the growth of the entire L1 ead.

To check whether excessive Wg also had any effect on ead cells, we overexpressed Wg by culturing *wg* ^*en-11*^, *UAS-GFP/CyO; hs-wg dpp-Gal4* at 30^°^C for 1 hr during the L1 stage. Such treatment did not elicit any detrimental effect on the ventral dpp-Gal4^+^ or dorsal wg-LacZ^+^ cells, indicating that these cells are sensitive to the loss of Wg but not to the gain of Wg. In contrast, the regions containing the midline cells and the ventral wg-LacZ^+^ cells were lost or reduced, indicating that growth of these cells is compromised by the high level of Wg (Fig. 3C-E). Thus, our data demonstrated that different types of cells in early ead react differently to the level of Wg for their growth.

### Hh is essential for the establishment of ventral domain

**Fig 4:**
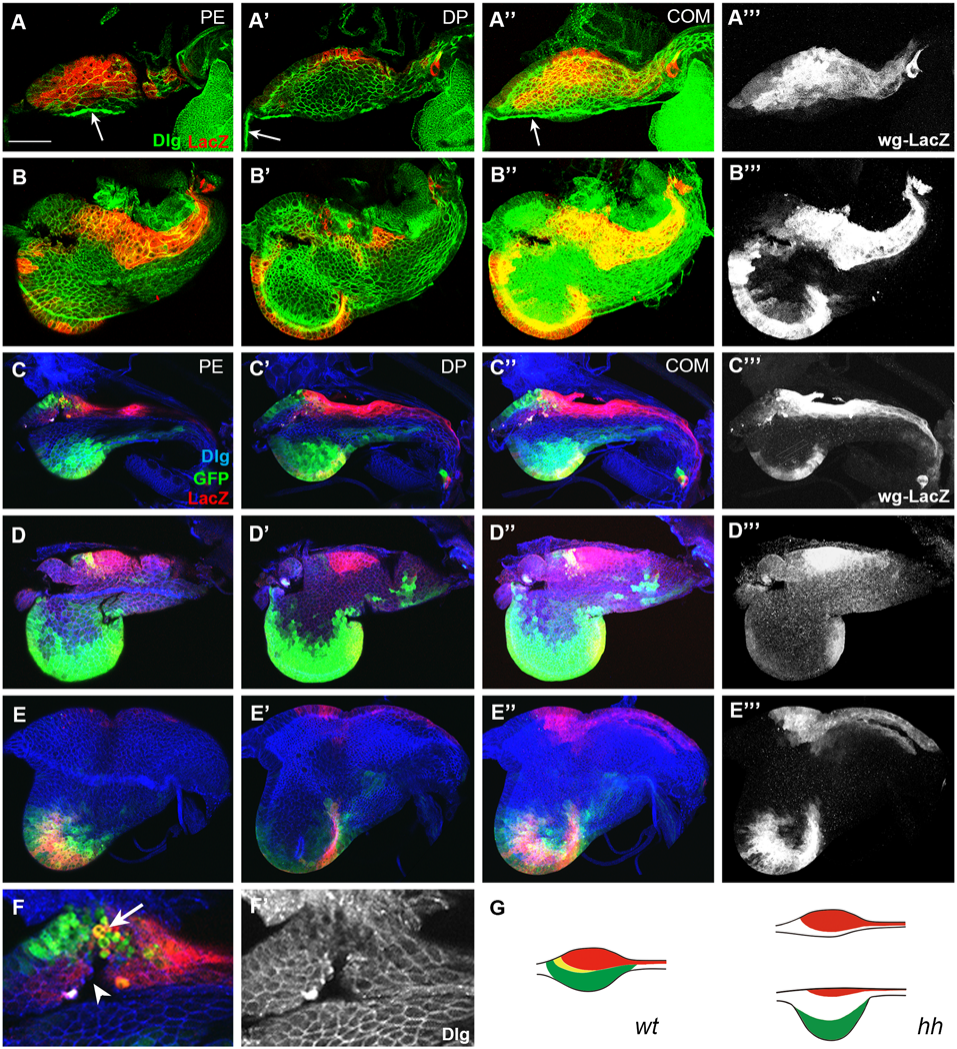
Domain-specific effects of *hh* mutation in eads. (A) An early L2 *hh* ^*ts2*^ disc without ventral domain. Bolwig’s nerve is marked with an arrow. (B) An early L3 ead with wg-LacZ^+^ cells at the entire ventral margin. (C) A late-L2 *hh* ^*ts2*^ ead with small dorsal domain without midline cells. (D) An early L3 ead without dorsal posterior domain in contrast to large ventral domain. (E) A late L3 ead without any dorsal dpp-gal4^+^ cells and only a small number of dorsal wg-LacZ^+^ cells. (F) Magnified image of C showing the damaged dorsal tissue (arrowhead) with round-shaped cells (arrow). (G) A diagram of *hh* ^*ts2*^ eads, one without the ventral domain, the other with large ventral domain. Scale bar: A, 20 μm; B, C, D, 30 μm; E, 100 μm; F, 10 μm.

To examine how Hh affects growth and patterning of the L1 ead, we checked the wg-LacZ pattern in *wg* ^*en-11*^ */CyO*; *hh* ^*ts2*^ */ hh* ^*ts2*^ eads as described in Materials and Methods. Two distinct phenotypes were observed in homozygous *hh* ^*ts2*^ eads. While ~30% (10 out of 32) of *hh* ^*ts2*^ eads had no ventral domain (Fig. 4A), the 70% (22 out of 32) of *hh* ^*ts2*^ eads had large ventral domains with small dorsal domains (Fig. 4B). These two opposing phenotypes were puzzling, because the loss of the ventral domain in *hh* ^*ts2*^ eads suggested that the initial formation of the ventral domain required Hh function, but the enlarged ventral domain in other *hh* ^*ts2*^ eads raised a possibility that the subsequent growth of the ventral domain may not require Hh. To test these possibilities, we cultured *hh* ^*ts2*^ embryos for one or two days at room temperature before the temperature shift and compared the frequency of the *hh* ^*ts2*^ eads that lacked the ventral domain. When the embryos were cultured for one day before the shift, ~40% (7 out of 17) of *hh* ^*ts2*^ eads were lacking ventral domain, but only ~10% (2 out of 18) were lacking ventral domain when they were cultured for two days before the shift. This implied that *hh* ^*ts2*^ eads with no ventral domains might have been shifted to restrictive temperature before the formation of ventral domain. These data support Hh as an important player for the establishment but not for subsequent growth of ventral domain. The effect of *hh* mutation on the patterning of L2 ead is summarized in Fig. 4G.

### Hh is required for cell division of dorsal cells

To study which cell types are regulated by Hh, we examined *wg* ^*en-11*^ */+*; *hh* ^*ts2*^ *UAS-GFP/ hh* ^*ts2*^ *dpp-Gal4* eads. As shown in Fig. 4C, the number of midline and dorsal dpp-Gal4^+^ cells on PE were reduced in L2 *hh* ^*ts2*^ eads. These PE cells were barely detectable in the early L3 *hh* ^*ts2*^ ead (Fig. 4D), and almost disappeared in the late L3 *hh* ^*ts2*^ ead (Fig. 4E). Similar to Fig. 4B, there were holes in the dorsal anterior region where the dorsal dpp-Gal4^+^ cells are normally present (Fig. 4C, D). Dorsal dpp-Gal4^+^ and wg-LacZ^+^ cells in the DP also showed similar phenotype (Fig. 4C’, D’). Round cells detached from the neighboring cells were often detected (Fig. 4F). We also observed that antenna disc was not formed in *hh* ^*ts2*^ eads (compare Fig. 4E to 2H). These data indicate that Hh is essential for cell division or survival of the dorsal cells of ead. In contrast, ventral dpp-Gal4^+^ cells and wg-LacZ^+^ cells in both PE and DP were present in L2 and L3 *hh* ^*ts2*^ eads, suggesting that cell division of ventral cells is not significantly affected by *hh* mutation (Fig. 4C-E).

**Fig 5:**
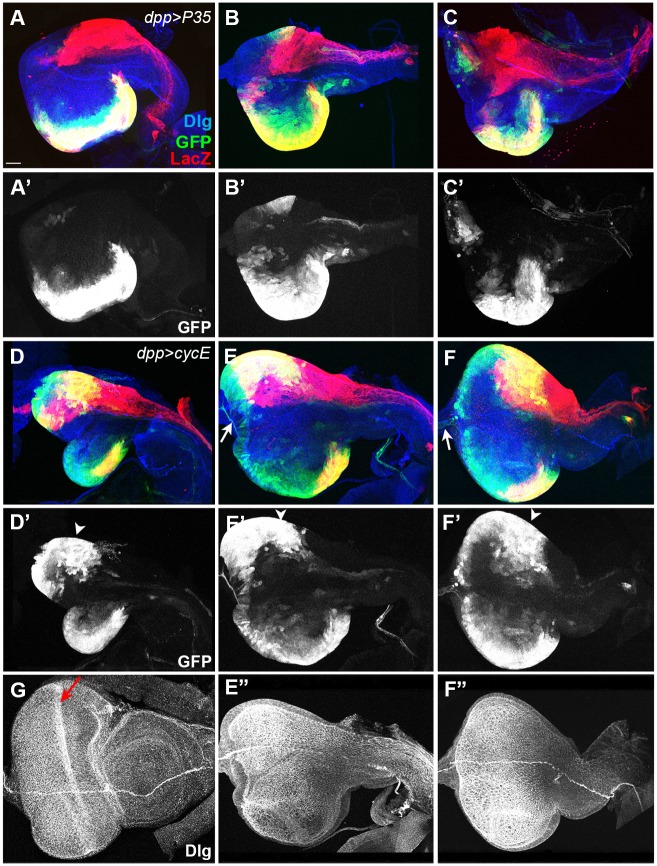
Expression of CycE but not P35 restores the balanced growth of *hh* ^*ts2*^ ead. (A-C) P35 expression by the *dpp-Gal4* driver in *hh* ^*ts2*^ eads had no effect on the *hh* ^*ts2*^ ead. (D-F) CycE expression by the *dpp-Gal4* driver in *hh* ^*ts2*^ eads restored dorsal dpp-Gal4^+^ cells (arrowheads). E” and F” are Dlg patterns of the eads shown in E and F. (G) Dlg pattern of the wild-type late L3 ead shown in Fig. 2I. Morphogenetic furrow is marked with a red arrow. Scale bar, 40 μm.

Hh is known to promote cell division by inducing the synthesis of Cyclin D and Cyclin E (CycE) [42]. Therefore, loss of dorsal cells observed in the *hh* ^*ts2*^ ead might be due to the lack of cell division. Alternatively, Hh might be required for the survival of these cells, as extensive cell death was previously observed in *hh* ^*ts2*^ ead [43]. To distinguish these two possibilities, we expressed P35 or CycE using the *dpp-Gal4* driver in *hh* ^*ts2*^ background because the two cell types lost in *hh* ^*ts2*^ ead were dorsal dpp-Gal4^+^ cells and midline cells in which the *dpp-Gal4* enhancer was active (Fig. 2). P35 expression in dpp-Gal4^+^ cells did not change the phenotype of *hh* ^*ts2*^ ead (Fig. 5A-C), but CycE expression in dpp-Gal4^+^ cells significantly increased the number of dorsal dpp-Gal4^+^ and midline cells (Fig. 5D-F). Therefore, Hh’s function for these dorsal cells in early larval stages is mainly to promote cell cycle for proliferation. Cell damages observed in *hh* ^*ts2*^ ead seemed to be a secondary effect stemmed from reduced cell division. Photoreceptors did not form in these discs (compare Fig. 5E”, F” to 5G), suggesting that expression of CycE by Hh can rescue cell division defect, but is not sufficient for the differentiation of photoreceptors [43,44].

### Hh regulates the expression from two *dpp* enhancers in the ventral domain

**Fig 6:**
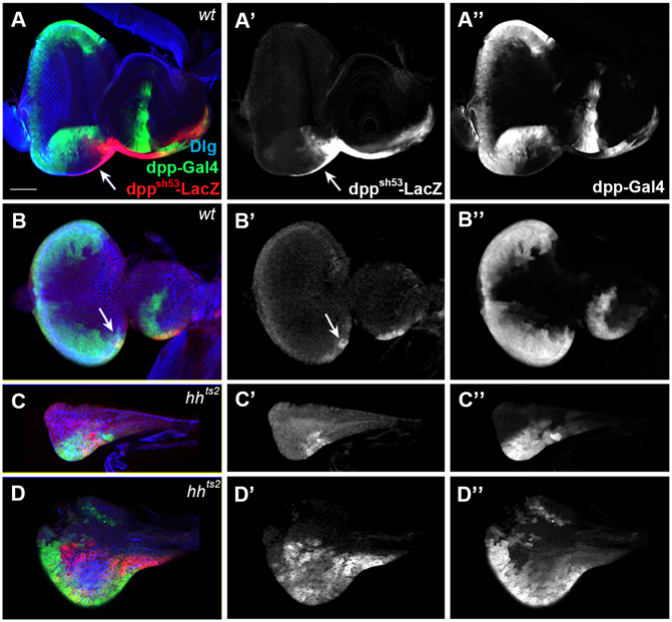
Hh regulates enhancer-specific *dpp* expression. (A, B) SH53-LacZ is expressed in a long narrow strip in the ventral margin of an L3 ead (arrow in A). It started to be expressed in the early L3 ead in the ventral anterior region of eye disc (arrow in B). (C-D) Both dpp-Gal4^+^ and SH53-LacZ^+^ cells are widely distributed in the ventral domains of L2 (C) and L3 (D) *hh* ^*ts2*^ eads. Scale bar, 90 μm.

The shape of *hh* ^*ts2*^ ead became almost normal by expression of CycE (compare Fig. 5F to 4E), suggesting that the abnormal shape of *hh* ^*ts2*^ ead is due to unbalanced growth between dorsal and ventral domains by *hh* mutation. In addition to growth problem, we noticed that expression of *dpp* and *wg* in *hh* ^*ts2*^ ead is abnormal: stronger and broader wg-LacZ and dpp-Gal4 expressions in the ventral domain of *hh* ^*ts2*^ ead than that of wild-type ead (Fig. 4D, E). To test whether *hh* mutation causes overall changes in Dpp expression, we examined Dpp-expressing cells driven by another *dpp* enhancer in the ventral domain. It has been shown that a *dpp* enhancer, *dpp* ^*sh-c*^, induces Dpp expression in a group of PE cells at the ventral margin in the late L3 ead [45], and we obtained the same result (Fig. 6A, A’). We also found that SH53-LacZ driven by *dpp* ^*sh-c*^ was not expressed in L1 and L2 wild-type eads, but detected from the early L3 stage at a very low level in the posterior ventral margin area (Fig. 6B, B’). The two enhancers, *dpp* ^*sh-c*^ and *dpp-Gal4*.*PS*, were rarely active in the same cells in wild-type L3 eads as shown by the absence of dpp-Gal4 expression in the cells expressing the SH53-lacZ reporter (Fig. 6A, A’). In contrast, the number of SH53-lacZ^+^ cells was significantly increased in *hh* ^*ts2*^ eads, and many cells expressed both dpp-Gal4 and SH53-LacZ markers (compare Fig. 6C, D to Fig. 6A, B). Thus, Hh seems to be important for regulation of *dpp* expression from the *dpp* enhancers in the ventral cells.

### Dpp is not important for the formation of ventral domain in the L1 ead but for the growth of ead in subsequent stages

**Fig 7:**
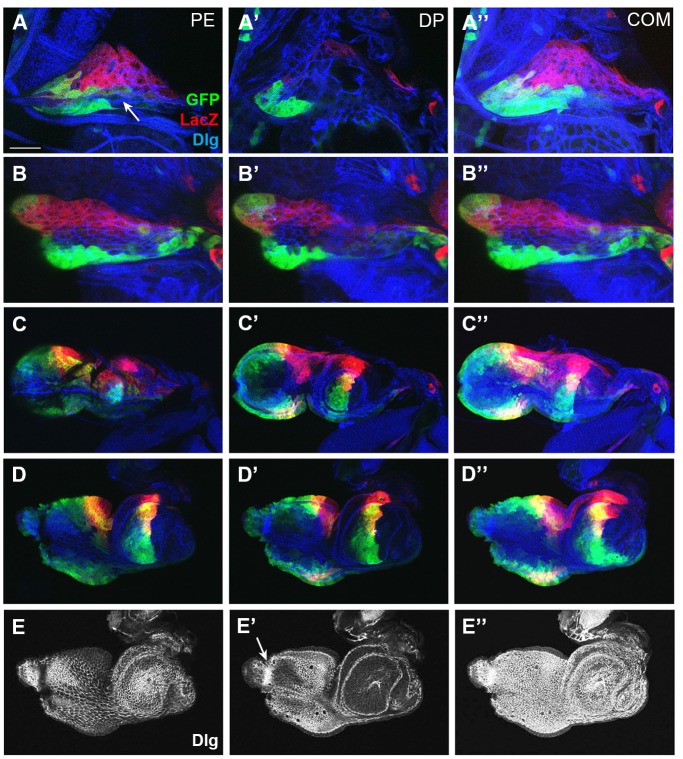
Dpp is important for the growth of ventral domain and eye disc. (A) The L1 *dpp* ^*d-blk*^ */dpp* ^*d14*^ ead shows normal pattern of dpp-Gal4 and wg-LacZ expression. (B) The ventral domain of L2 *dpp* mutant ead was smaller than wild-type (compare to Fig. 2E). (C) The *dpp* mutant ead with smaller eye part than wild-type (compare to Fig. 2I). (D) The L3 *dpp* mutant ead with minimized eye field. The arrow points to the morphogenetic furrow (middle panel). (E) The images of Dlg in (D) show the cell boundary. Scale bar: A, 12 μm; B, μm; C, D 90 μm.

To examine which cell types are affected in *dpp* mutants, we reduced Dpp function using a heteroallelic combination of *dpp* ^*d-blk*^ */dpp* ^*d14*^ [46,47]. Cells ventral to the Bolwig’s nerve in the L1 *dpp* ^*d-blk*^ */dpp* ^*d14*^ ead expressed dpp-Gal4 at a high level, demonstrating that the induction of *dpp* transcription occurred normally even when Dpp function is compromised (Fig. 7A). However, the L2 *dpp* mutant ead was thinner than the wild-type L2 ead, and its ventral domain that contains mostly dpp-Gal4^+^ cells was smaller than the dorsal domain that contains mostly wg-LacZ^+^ cells (compare Fig. 7B to Fig. 2E). Similarly, both dorsal and ventral dpp-Gal4^+^ cells in the L3 *dpp* mutant eye disc were significantly reduced in number (Fig. 7C). Photoreceptors were hardly found in all discs examined, as addressed by the previous reports that Dpp is essential for retinal differentiation [7,44].

## Discussion

We have previously shown that Wg, Hh, and Dpp play essential roles in growth and patterning of the ead during the L1 stage [11]. In this study, we further examined the pattern of both wg-LacZ and dpp-Gal4 markers in eads from hatching to the L3 stage. These two markers were used not only for marking cells expressing Wg and Dpp but also for identifying specific cell types located at distinct regions of PE and DP of eads. Examination of these two markers in both wild type and mutants allowed us to study the growth and patterning of ead.

An unexpected finding in this study was that the L1 ead right after hatching has only the dorsal domain expressing Wg. Subsequently, induction of *dpp* transcription in a few cells along the Bolwig’s nerve coincided with the formation of the ventral domain. This induction of *dpp* transcription took place in neither *wg* nor some *hh* mutant eads, suggesting that both Wg and Hh are required for induction of *dpp* expression. Therefore, Hh and Wg act upstream of Dpp in L1 ead. Requirements of Wg and Hh for *dpp* expression have been identified in other developmental processes such as denticle patterning in the embryonic epidermis [48,49] and ocelli development [50]. After the formation of the ventral domain, Dpp was required for growth and differentiation of eye disc as shown in Fig. 7 and other reports [7,44].

Although both Wg and Hh are essential for induction of Dpp^+^ cells as an initial step for formation of ventral domain, there is a critical difference between Wg and Hh on the subsequent growth of ventral domain. Wg was important for the growth of the ventral domain, but Hh was important for the regulation of *dpp* induction but not for the cell division of the ventral domain. Therefore, Wg seems to act for the growth of ventral ead as a mitogen, while Hh differentially regulates *dpp* transcription as also observed in wing discs [51–53]. This demonstrates that Hh’s role is different in the two domains: it acts for cell division in the dorsal domain, and for regulation of *dpp* transcription in the ventral domain.

Cells on the ventral side of the Bolwig’s nerve first appeared in the mid-L1 ead, and the ventral domain is finally formed in the mid-L2 ead. We propose that cells with multiple origins grow together during this critical period of ventral formation based on two observations. First, both dpp-Gal4^+^ and dpp-Gal4^-^ cells are present in the established ventral domain of mid-L2 eads. Second, multiple GFP clones with different shapes and positions were repeatedly observed in the ventral PE. Where did these ventral cells come from? One of them is the dpp-Gal4^+^ cells on the ventral side of the Bolwig’s nerve as shown in Fig. 1. Other cells may be hiding on the ventral side of the Bolwig’s nerve without cell division and then start to divide at the onset of ventral formation. The GFP clones generated in the ventral DP were more difficult to interpret than their counterparts in PE due to their complexity and irregularity (data not shown), suggesting that the ventral DP also has multiple embryonic origins as well. Identification of cellular origin in ventral domain may be crucial for understanding the growth and patterning of L1 ead.

The Bolwig’s nerve in early ead projects along the ventral margin of L1 ead prior to the growth of the ventral domain, and then changes its position to an approximate midline as the ventral domain of ead further expands in the following larval stages. Although the Bolwig’s nerve does not strictly coincide with a clonal boundary, its projection pattern in early ead makes it as an appropriate DV marker for PE. It is important to point out that the Bolwig’s nerve does not reflect the equatorial midline of DP defined by the DV planar polarity, despite Bolwig’s nerve has been occasionally used as a convenient marker for DV midline of wild-type L3 ead [7].

We have previously reported that expression of Wg and Hh in PE cells are able to induce changes in underlying DP cells [11]. Another way of PE cells to influence DP cells is to become special DP cells that retain the properties of PE cells. For example, polar margin DP cells that are originated from PE influence the planar polarity of neighboring photoreceptor cells [54,55]. These Wg-secreting margin cells may be clonally related to the dorsal wg-LacZ^+^ cells in L1 ead described here (Fig. 1). In addition to planar cell polarity, Wg secreted from these margin cells is involved in the formation of specific dorsal eye structure called dorsal rim ommatidia (DR) that are specialized for detecting polarized light [56,57]. Besides the margin cells involved in Wg signaling, other posterior margin cells are shown to secret Hh to induce retina differentiation [9]. Hh^+^ clones generated in these posterior margin DP cells are able to induce expression of Serrate in the neighboring DP cells [11]. These margin cells are clonally related to the PE cells, and that is why the margin DP cells behave differently from other DP cells. Consistent with these findings, *patched* mutant clones generated in the margin behave differently from those generated in other parts of the eye field in affecting planar cell polarity of photoreceptor cells [58]. Therefore, DP cells originated from PE cells still maintain the property of PE and play important roles in differentiation of retina.

Distinct cell types in the L1 ead become specified by cooperative or antagonistic interaction between multiple proteins, and this specification is prerequisite for the formation of multiple organs from the ead during later stages [59]. Since ead is composed of six embryonic head segments [17,23,24], the distinct cell types are most likely originated from different embryonic segments. When the location of the six embryonic segments in the L1 ead is identified, it will become possible to link the embryonic ead to cell types in larval ead and ultimately to adult head structures. By the same token, it will be possible to identify the cell types that are responsible for the formation of retina. Combination of clonal analysis in both PE and DP layers, expression pattern of various proteins, and mutant analysis will help understand how the complicated adult head structures are generated from a small disc with only ~20 cells at the beginning.

## Supporting Information

S1 Fig The wg-LacZ reporter in *wg* ^*en-11*^ allele reflected the level of Wg protein. Anti-Wg antibody (4D4) and wg-LacZ^+^ cells are marked with green and red, respectively. The vesicular forms of Wg is highest in wg-LacZ^+^ cells and the level of Wg decreased as the distance from the wg-LacZ^+^ cells increased in both late L2 (A) and late L3 (C) *wg* ^*en-11*^ */CyO* ead. Scale bar, 90 μm.
(TIF)

S2 Fig Two nerve-like structures on the ventral side of the early L1 ead. (A-E) Serial section images of an early L1 ead. All images were serially captured from PE layer (A) to DP layer (E) with 1 μm interval. A is the combined image of B to E. White arrow and yellow arrow mark the Bolwig’s nerve and the optic stalk, respectively. (B) was used in Fig. 1D. (F-G) Image in the Fig. 1D was magnified 2 times in PE (F) and DP (G) layers. Arrow and arrowhead indicate Bolwig’s nerve and the unknown nerve, respectively. Yellow arrow points the unknown nerve entering the brain. Scale bar: A-E, 20 μm; F-G, 10 μm.
(TIF)

S3 Fig Serial images of Fig. 1E. All images were serially captured from PE layer (B) to DP layer (J) with 0.63 μm interval. A is a combined image. Scale bar, 40 μm.
(TIF)

S4 Fig Serial images of Fig. 1F. All images were serially captured from PE layer (B) to DP layer (O) with 1 μm interval. A is a combined image. btl, breathless. Scale bar, 10 μm.
(TIF)

S5 Fig Serial images of Fig. 1H. All images were serially captured from PE layer (B) to DP layer (O) with 1.5 μm interval. A is a combined image. Scale bar, 10 μm.
(TIF)

S6 Fig Serial images of Fig. 1J. All images were serially captured from PE layer (B) to DP layer (O) with 1.5 μm interval. A is a combined image. Scale bar, 10 μm.
(TIF)

S7 Fig Serial images of Fig. 2A. All images were serially captured from PE layer (B) to DP layer (O) with 1.5 μm interval. A is a combined image. Scale bar, 20 μm.
(TIF)

S8 Fig Dorsal PE cells are clonally distinct from the ventral PE cells in ead. All GFP clones in the PE are consistently generated in anterior-posterior direction. (A-D) Bolwig’s nerve is a domain boundary but not a clonal boundary in PE. (A, C) Two eads with GFP clones in the PE of an L3 ead. Images of the white-boxed region in (A) and (C) are magnified in (B) and (D), respectively. Yellow arrows indicate midline GFP clones that trespass on Bolwig’s nerve marked by white arrows. (B’) and (D)’ are black and white images of Dlg pattern with Bolwig’s nerve marked by blue line. (E) Multiple clones in another ead. (F, G) GFP clones were restricted in either ventral (F) or dorsal (G) domain in the PE. Scale bar, 50 μm.
(TIF)

S9 Fig Serial images of Fig. 2H. All images were serially captured from PE layer (B) to DP layer (O) with 1.5 μm interval. A is a combined image. Scale bar, 20 μm.
(TIF)

S10 Fig Dorsal wg-LacZ^+^ cells may be clonal. (A) Two independent GFP clones in the PE of an L3 ead. Strong LacZ signal is from the DP, and weak LacZ expression in the PE is not obvious in this image. (B) Image of the boxed region in A was taken at the PE level. These two independent GFP clones (arrow and arrowhead) are next to the wg-LacZ^+^ cells. (C, D) A GFP clone is juxtaposed to wg-LacZ^+^ cells in the PE of the folded region that is continuous from the dorsal PE (arrowhead), and its magnified image is shown in D. Scale bar: A, C, 145 μm; B, 30 μm; D, 25 μm.
(TIF)
