# Correction: Cell Type-Specific Responses to Wingless, Hedgehog and Decapentaplegic Are Essential for Patterning Early Eye-Antenna Disc in Drosophila

**DOI:** 10.1371/journal.pone.0128169

**Published:** 2015-05-08

**Authors:** Jong-Hoon Won, Orkhon Tsogtbaatar, Wonseok Son, Amit Singh, Kwang-Wook Choi, Kyung-Ok Cho

The second author’s name is spelled incorrectly. The correct name is: Orkhon Tsogtbaatar. The correct citation is: Won J-H, Tsogtbaatar O, Son W, Singh A, Choi K-W, Cho K-O (2015) Cell Type-Specific Responses to Wingless, Hedgehog and Decapentaplegic Are Essential for Patterning Early Eye-Antenna Disc in Drosophila. PLoS ONE 10(4): e0121999. doi:10.1371/journal.pone.0121999
