# Supplementary figures and images for: Cell Type-Specific Responses to Wingless, Hedgehog and Decapentaplegic Are Essential for Patterning Early Eye-Antenna Disc in Drosophila
Source: PLoS One. 2015 Apr 7;10(4):e0121999. doi: 10.1371/journal.pone.0121999 (PMC4388393; doi:10.1371/journal.pone.0121999)

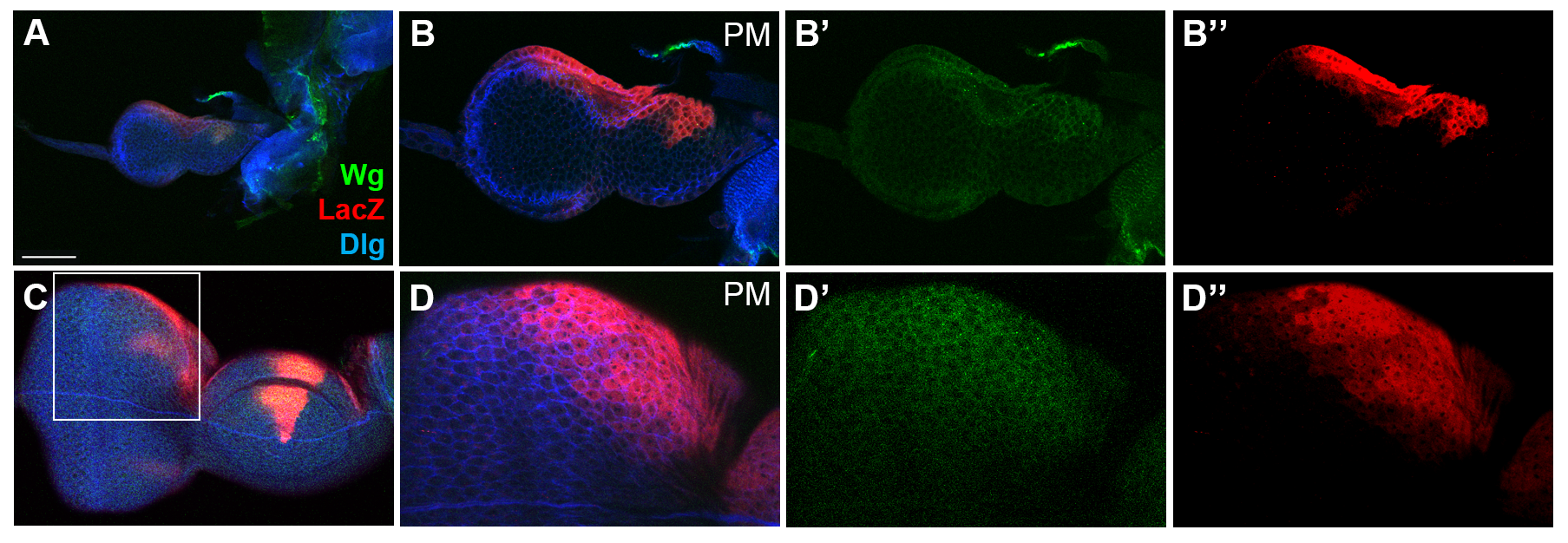

Supplement: S1 Fig — Anti-Wg antibody (4D4) and wg-LacZ+ cells are marked with green and red, respectively. The vesicular forms of Wg is highest in wg-LacZ+ cells and the level of Wg decreased as the distance from the wg-LacZ+ cells increased in both late L2 (A) and late L3 (C) wg en-11 /CyO ead. Scale bar, 90 μm. (TIF) [file pone.0121999.s001.tif]

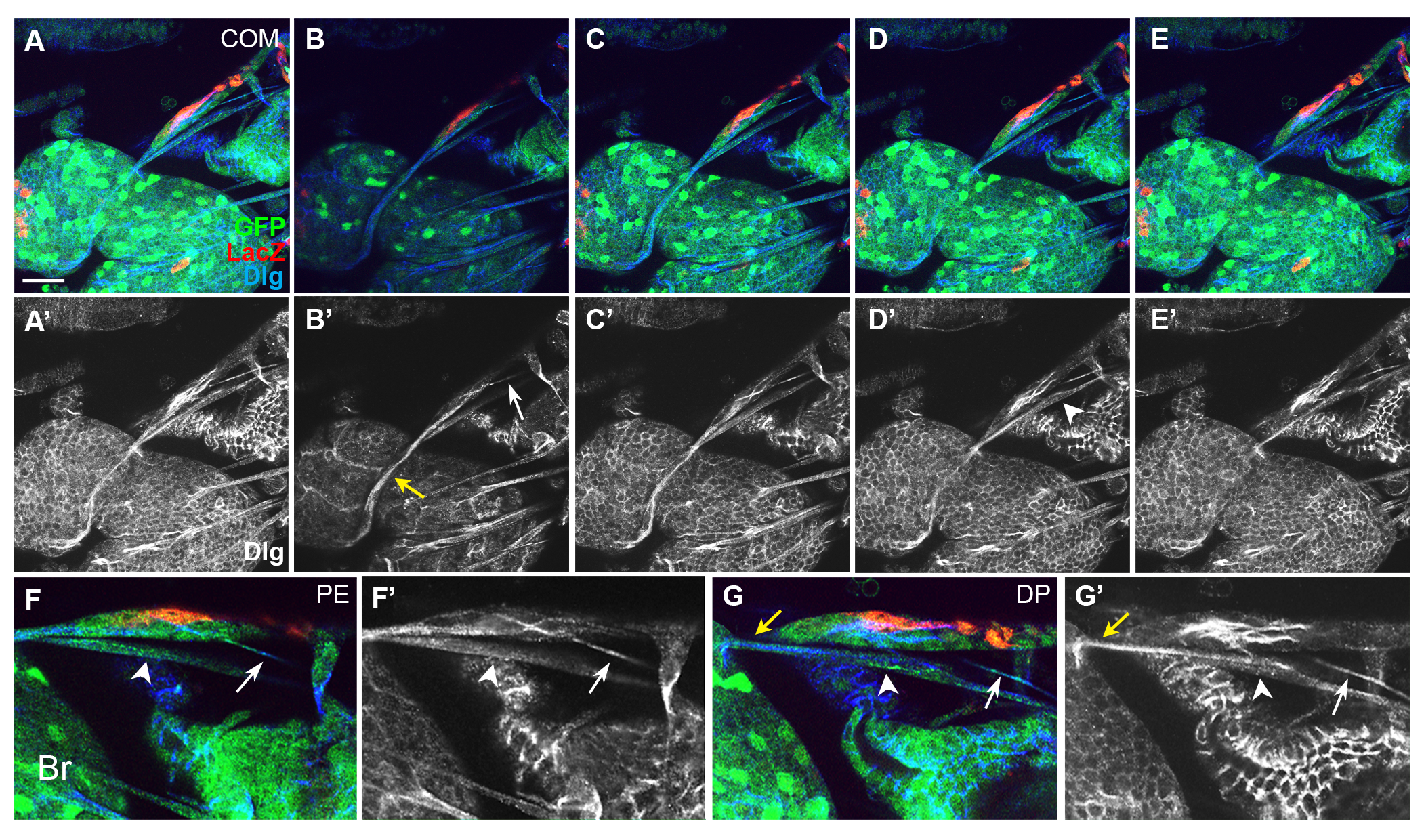

Supplement: S2 Fig — (A-E) Serial section images of an early L1 ead. All images were serially captured from PE layer (A) to DP layer (E) with 1 μm interval. A is the combined image of B to E. White arrow and yellow arrow mark the Bolwig’s nerve and the optic stalk, respectively. (B) was used in Fig. 1D. (F-G) Image in the Fig. 1D was magnified 2 times in PE (F) and DP (G) layers. Arrow and arrowhead indicate Bolwig’s nerve and the unknown nerve, respectively. Yellow arrow points the unknown nerve entering the brain. Scale bar: A-E, 20 μm; F-G, 10 μm. (TIF) [file pone.0121999.s002.tif]

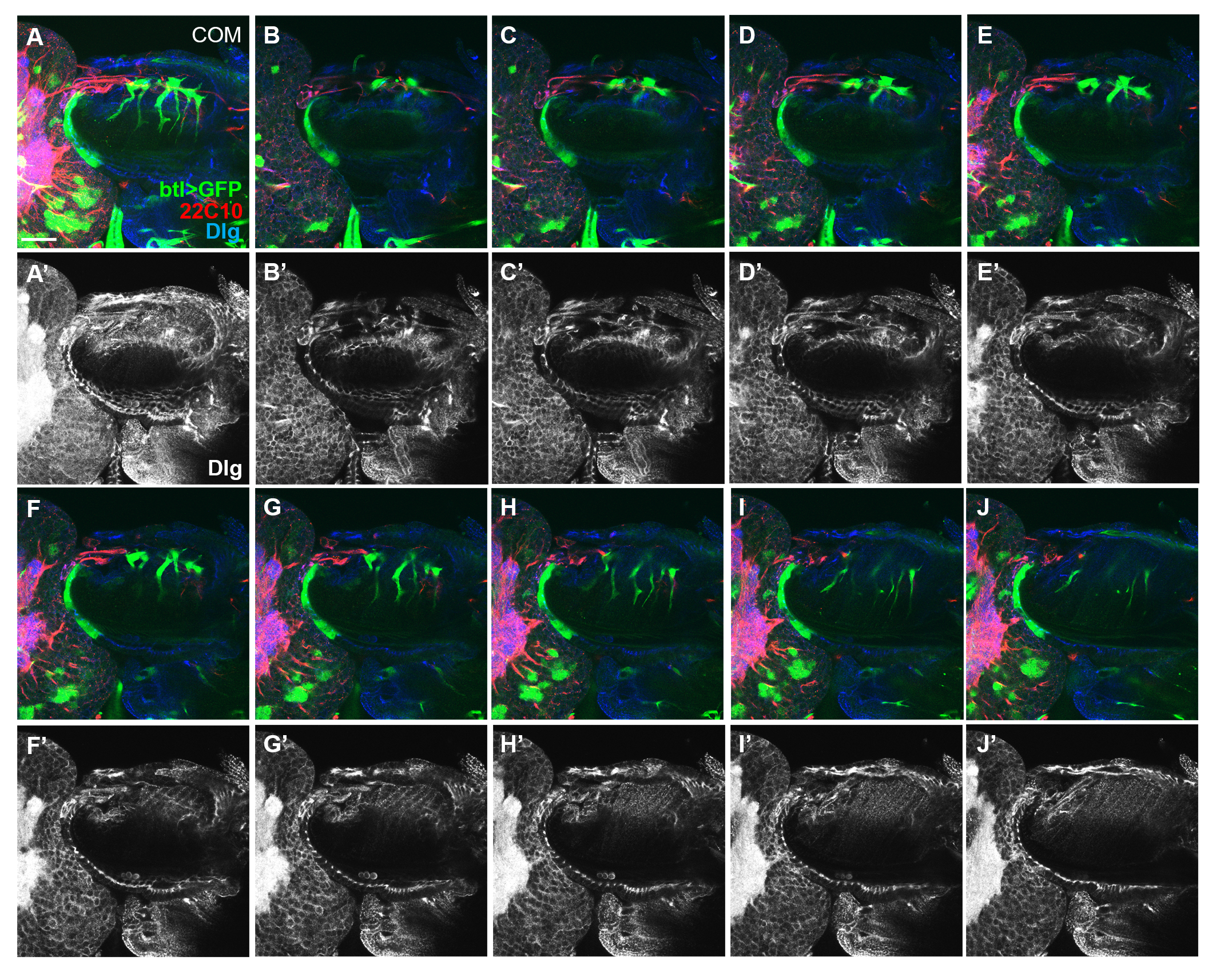

Supplement: S3 Fig — All images were serially captured from PE layer (B) to DP layer (J) with 0.63 μm interval. A is a combined image. Scale bar, 40 μm. (TIF) [file pone.0121999.s003.tif]

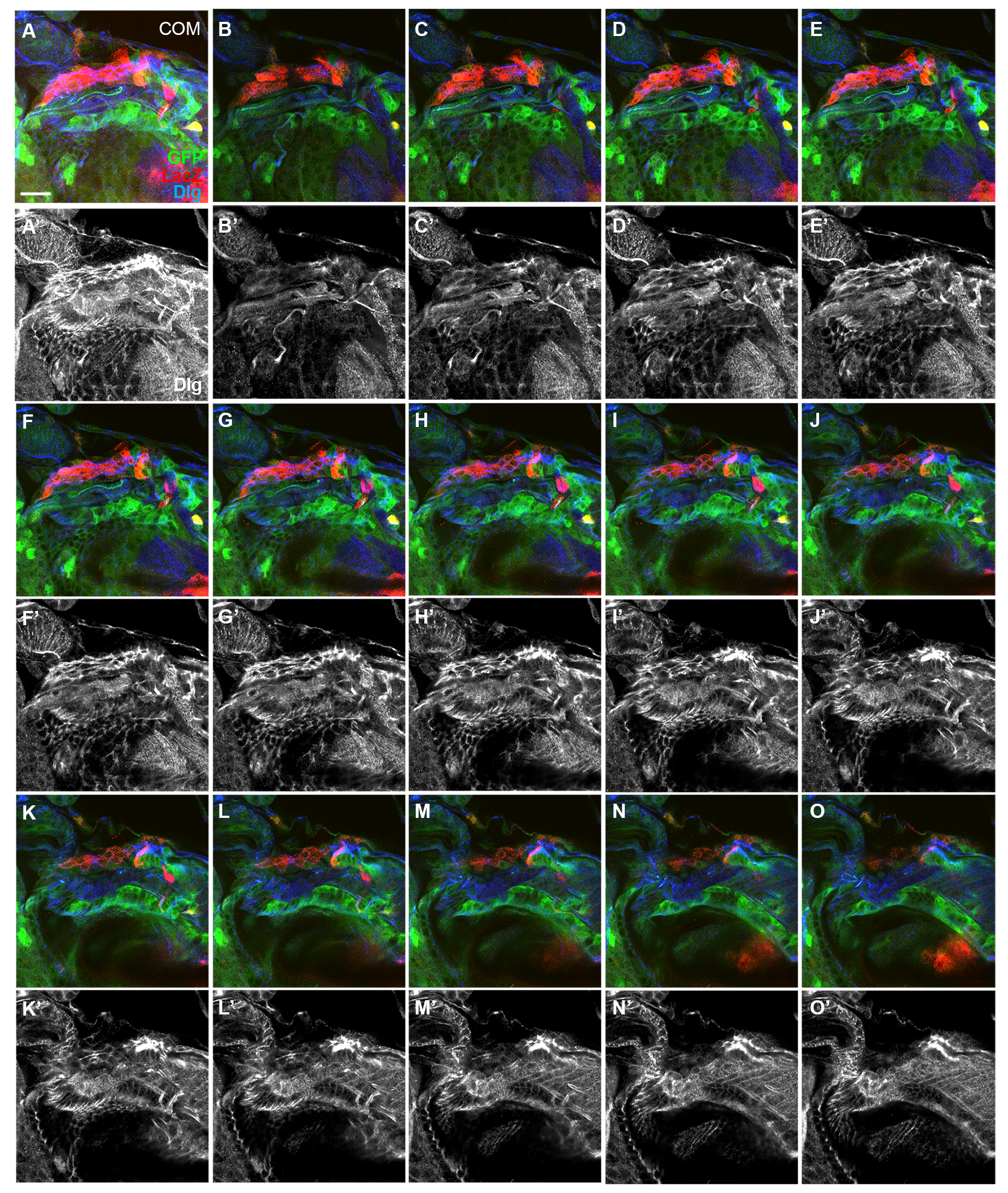

Supplement: S4 Fig — All images were serially captured from PE layer (B) to DP layer (O) with 1 μm interval. A is a combined image. btl, breathless. Scale bar, 10 μm. (TIF) [file pone.0121999.s004.tif]

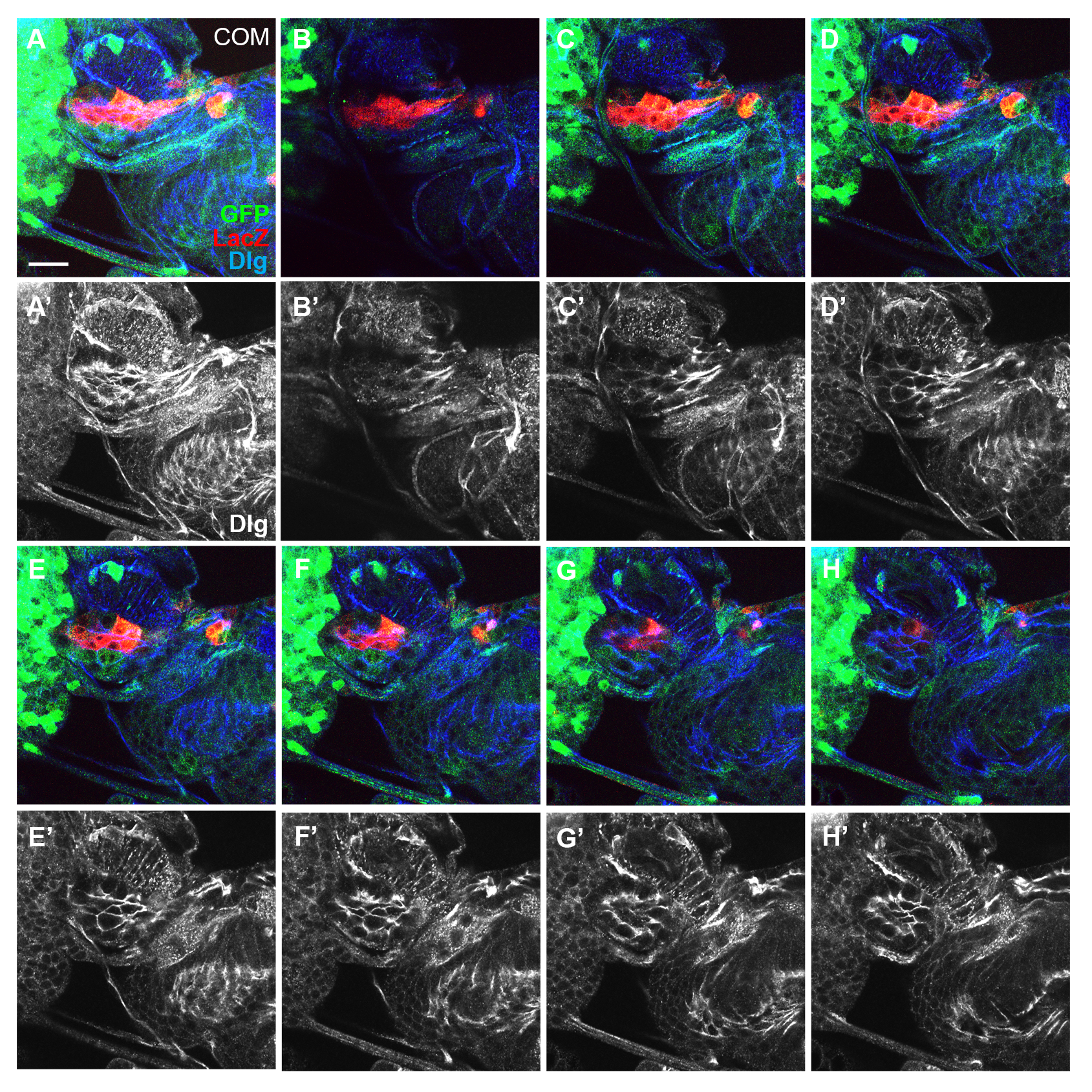

Supplement: S5 Fig — All images were serially captured from PE layer (B) to DP layer (O) with 1.5 μm interval. A is a combined image. Scale bar, 10 μm. (TIF) [file pone.0121999.s005.tif]

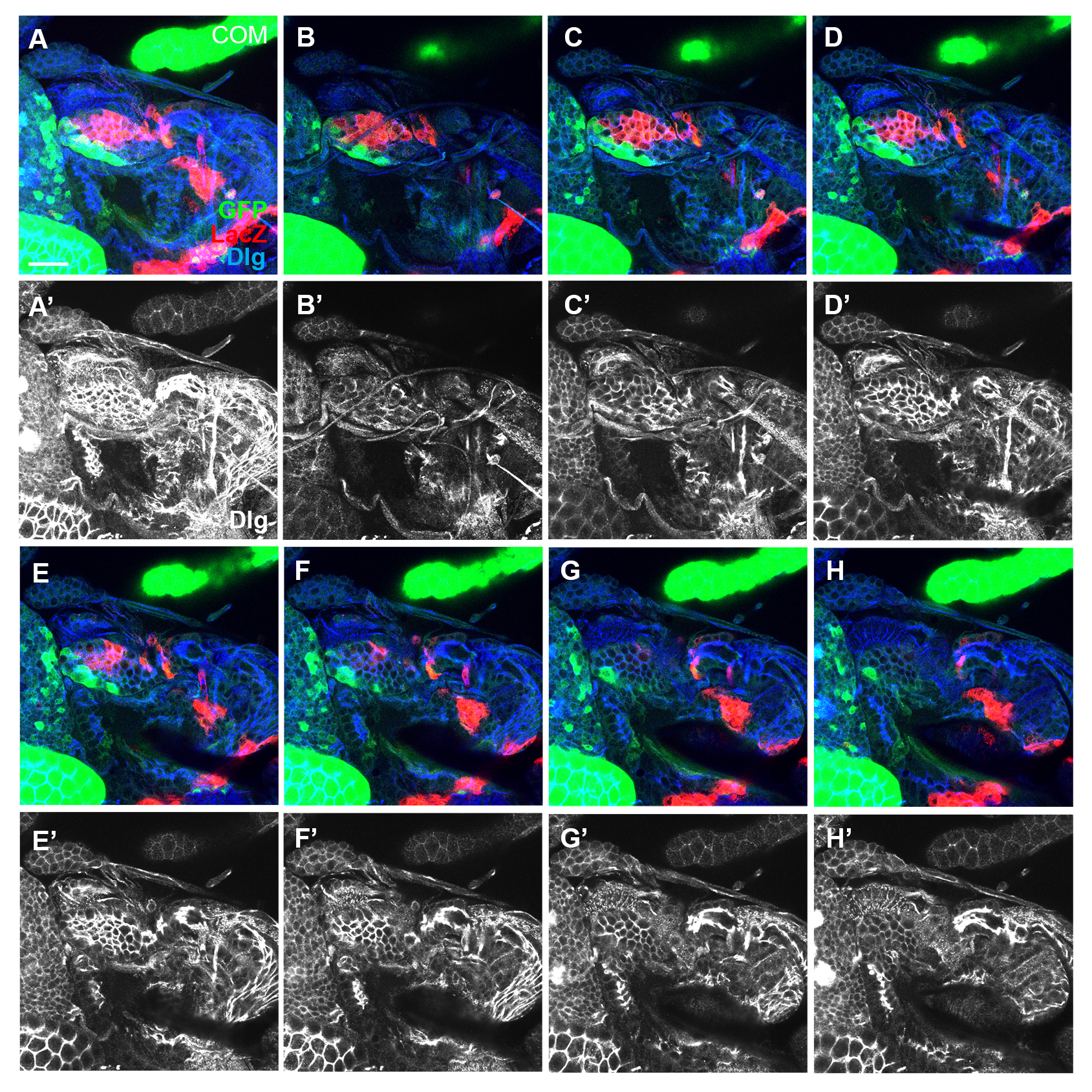

Supplement: S6 Fig — All images were serially captured from PE layer (B) to DP layer (O) with 1.5 μm interval. A is a combined image. Scale bar, 10 μm. (TIF) [file pone.0121999.s006.tif]

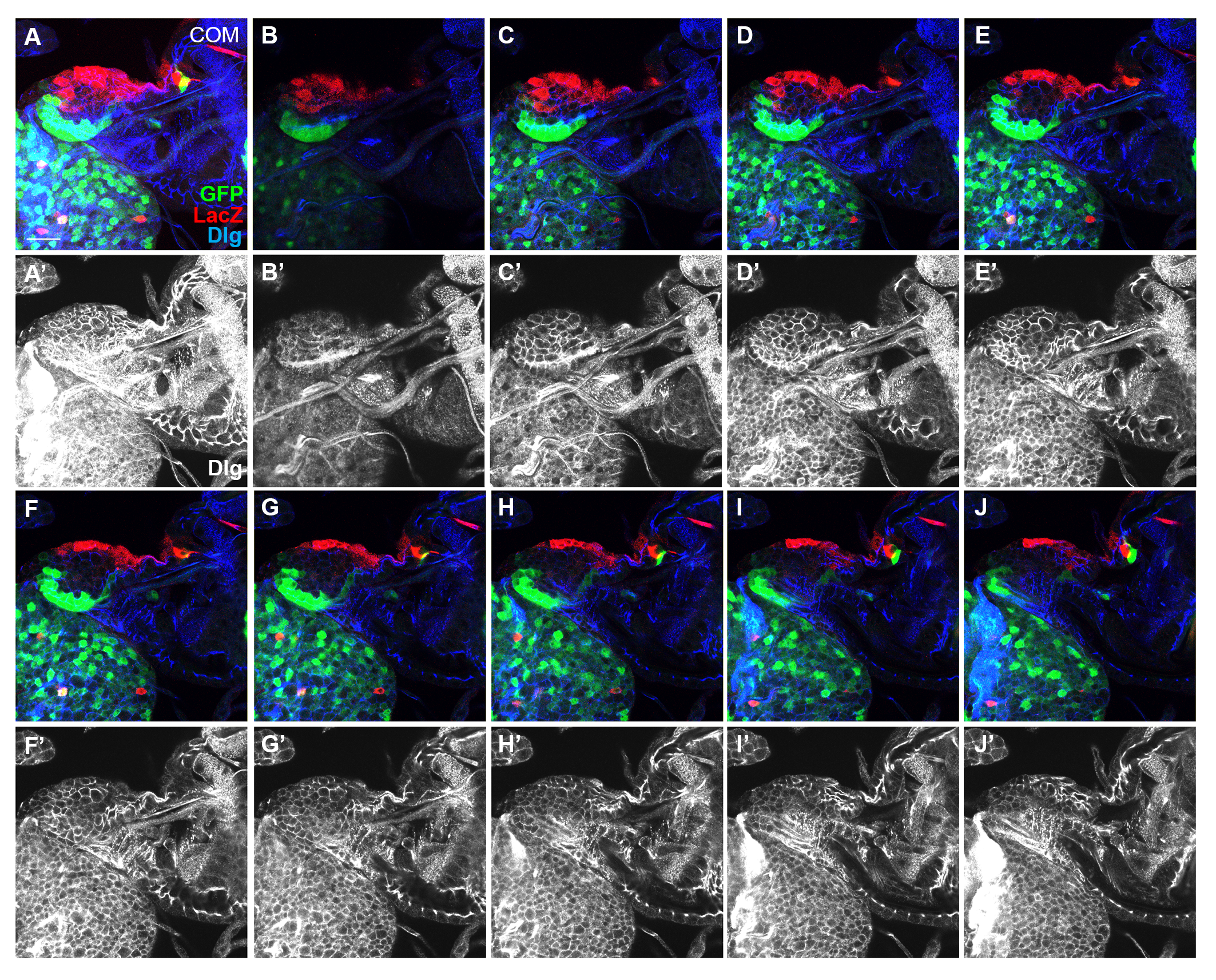

Supplement: S7 Fig — All images were serially captured from PE layer (B) to DP layer (O) with 1.5 μm interval. A is a combined image. Scale bar, 20 μm. (TIF) [file pone.0121999.s007.tif]

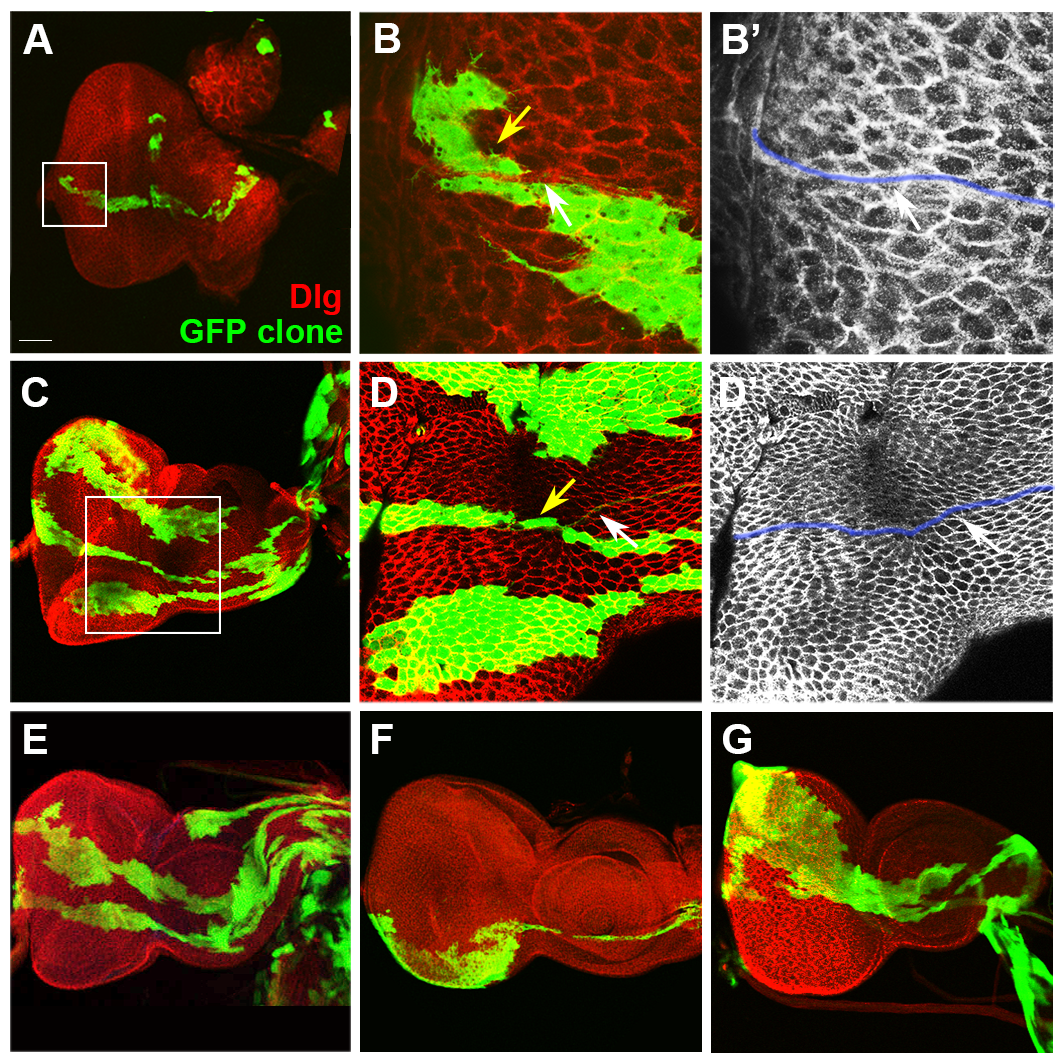

Supplement: S8 Fig — All GFP clones in the PE are consistently generated in anterior-posterior direction. (A-D) Bolwig’s nerve is a domain boundary but not a clonal boundary in PE. (A, C) Two eads with GFP clones in the PE of an L3 ead. Images of the white-boxed region in (A) and (C) are magnified in (B) and (D), respectively. Yellow arrows indicate midline GFP clones that trespass on Bolwig’s nerve marked by white arrows. (B’) and (D)’ are black and white images of Dlg pattern with Bolwig’s nerve marked by blue line. (E) Multiple clones in another ead. (F, G) GFP clones were restricted in either ventral (F) or dorsal (G) domain in the PE. Scale bar, 50 μm. (TIF) [file pone.0121999.s008.tif]

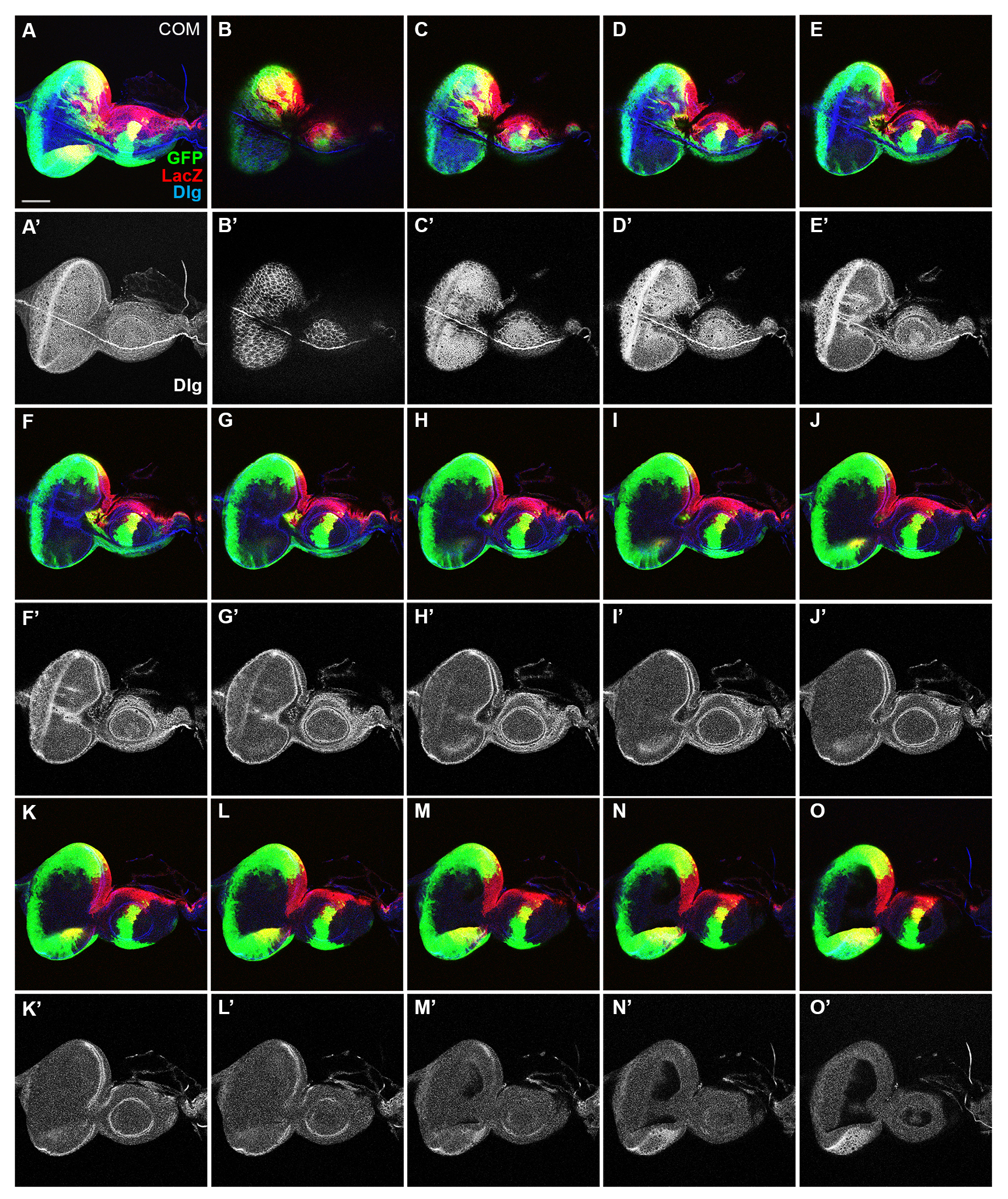

Supplement: S9 Fig — All images were serially captured from PE layer (B) to DP layer (O) with 1.5 μm interval. A is a combined image. Scale bar, 20 μm. (TIF) [file pone.0121999.s009.tif]

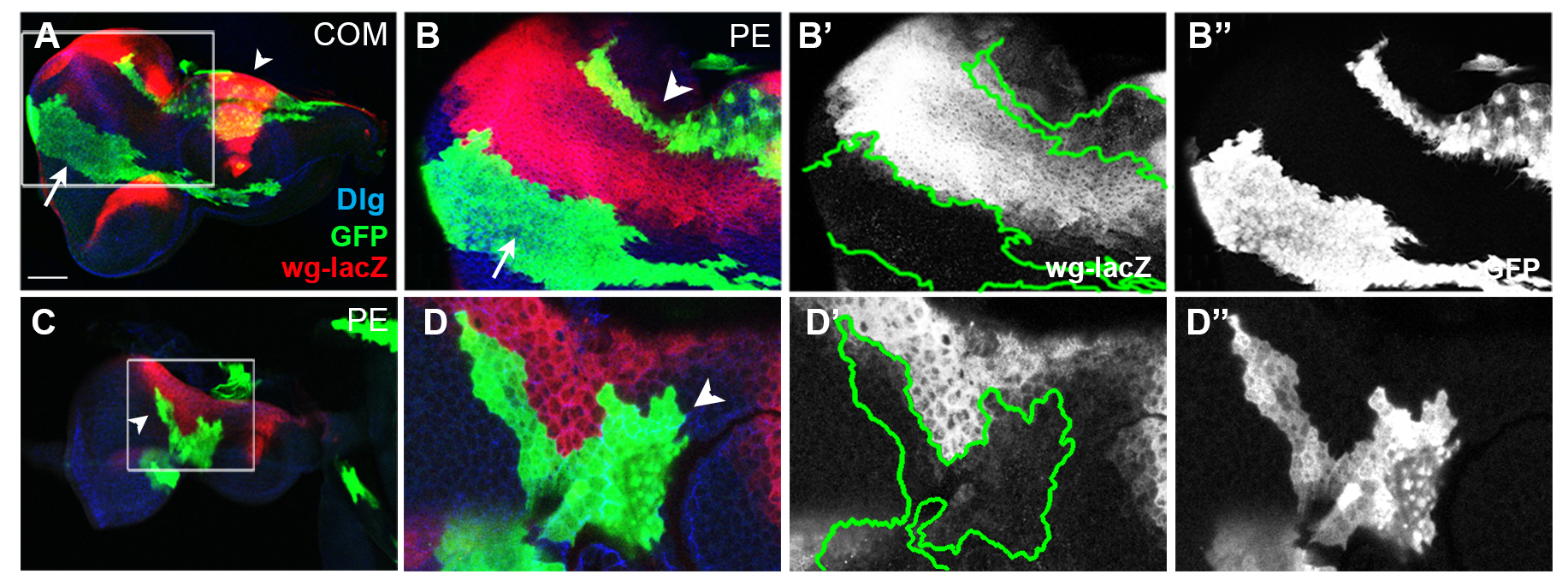

Supplement: S10 Fig — (A) Two independent GFP clones in the PE of an L3 ead. Strong LacZ signal is from the DP, and weak LacZ expression in the PE is not obvious in this image. (B) Image of the boxed region in A was taken at the PE level. These two independent GFP clones (arrow and arrowhead) are next to the wg-LacZ+ cells. (C, D) A GFP clone is juxtaposed to wg-LacZ+ cells in the PE of the folded region that is continuous from the dorsal PE (arrowhead), and its magnified image is shown in D. Scale bar: A, C, 145 μm; B, 30 μm; D, 25 μm. (TIF) [file pone.0121999.s010.tif]
